# Supplementary material for: Fragmented micro-growth habitats present opportunities for alternative competitive outcomes
Source: Nat Commun. 2024 Aug 31;15:7591. doi: 10.1038/s41467-024-51944-z (PMC11365936; doi:10.1038/s41467-024-51944-z)
Supplement: Supplementary file 1 — Supplementary information [file 41467_2024_51944_MOESM1_ESM.pdf]

SUPPLEMENTARY INFORMATION

Fragmented micro-growth habitats present opportunities for alternative competitive outcomes

Maxime Batsch[1], Isaline Guex[2], Helena Todorov[1], Clara M. Heiman[1], Jordan Vacheron[1], Julia A. Vorholt[3], Christoph Keel[1], and Jan Roelof van der Meer[1]

1) Department of Fundamental Microbiology, University of Lausanne, 1005 Lausanne, Switzerland

2) Department of Mathematics, University of Fribourg, Fribourg, Switzerland

3) Institute for Microbiology, Swiss Federal Institute of Technology (ETH Zurich), Zürich, Switzerland

Supplementary tables

Supplementary Table 1: Strain-specific fluorescence distributions of solo or mix droplets with *S. wittichii* RW1 and *Pseudomonas* sp. Leaf15 under substrate indifference conditions

Supplementary Table 2: Generalized linear mixed effects analysis for paired productivities of *P. putida* and *P. veronii* in timelapse imaged droplets

Supplementary Table 3: 21C growth medium composition

Supplementary Table 4: Strains and pre-culture procedures

Supplementary Table 5: Strains and media conditions for each experiment

Supplementary figures

Supplementary Fig. 1: Encapsulation of cells in picoliter droplets by microfluidic operation.

Supplementary Fig. 2: Starting cell distributions for *P. putida* and *P. veronii* after encapsulation in 35-pL volume droplets.

Supplementary Fig. 3: Growth (as increase of culture turbidity) of *P. putida* or *P. veronii* in liquid suspended monocultures.

Supplementary Fig. 4 : Comparison of T=0 and T24 or T48 h droplet AF Area x fluorescence levels for *P. putida* and *P. veronii*.

Supplementary Fig. 5 : Droplet growth outcomes in an isogenic *P. putida* co-culture control on 10 mM succinate.

Supplementary Fig 6: Growth curves of *P. putida* and *P. veronii* in liquid culture on D-mannitol and putrescine.

Supplementary Fig. 7: Growth curves of *Pseudomonas* L15 and *Sphingomonas wittichii* RW1 in liquid culture on succinate or salicylate.

Supplementary Fig. 8: Individual replicates of productivities of *Pseudomonas* Leaf15 and *Sphingomonas wittichii* RW1 in pL droplets sampled at different incubation times.

Supplementary Fig. 9: Stationary phase productivity of *P. putida* and *P. veronii* in mix droplets as a function of observed lag time.

Supplementary Fig. 10: Paired growth trajectories of *P. putida* and *P. veronii* in time-lapse imaged droplets.

Supplementary Fig. 11: Effects of droplet starting cell numbers on observed population growth kinetics.

Supplementary Fig. 12: Simulated effects of increasing starting cell numbers on *P. veronii* relative abundance at stationary phase (mix droplets only).

Supplementary Fig. 13: Measured per droplet starting cell distributions at higher intended founder cell population sizes of *P. putida* and *P. veronii*.

Supplementary Fig. 14: Strain-specific productivity scatter plots for mix droplets at increasing starting cell population sizes of *P. putida* and *P. veronii*.

Supplementary Fig. 15: Gating strategy for strain-specific cell detection in flow cytometry.

Supplementary Fig. 16: Distributions of growth kinetic parameters inferred from droplets with a single founder cell of *P. putida* or *P. veronii*.

Supplementary Fig. 17: Reproducibility of droplet cultivation procedures.

Supplementary references

Supplementary Table 1. Strain-specific fluorescence distributions of solo or mix droplets with *S. wittichii* RW1 and *Pseudomonas* sp. Leaf15 under substrate indifference conditions.

Comparison	*S. wittichii* RW1		*P*-value[1]	*Pseudomonas* sp. Leaf15		*P*-value
	Solo droplets	Mix droplets		Solo droplets	Mix droplets	
Mean of replicate medians [2]	0.8564	0.9079	0.0488	0.9306	0.9979	0.0019
Mean of replicate 90th percentile	1.8395	1.9058	0.1797	1.4989	1.6074	0.0019
Mean of replicate 10th percentile	0.3488	0.3322	0.2129	0.5437	0.5947	0.0273
Mean fraction of top-10 percentiles	0.0995	0.1158	0.125	0.1000	0.1325	0.0019
Mean fraction of low-10 percentiles	0.0995	0.1213	0.0820	0.1000	0.0837	0.0488

1) Two-sided Sign-rank test implemented in MATLAB.
2) Three biological replicates from three sampling time points combined

Supplementary Table 2. Generalized linear mixed effects analysis for paired productivities of *P. putida* and *P. veronii* in timelapse imaged droplets.

**Fixed effects coefficients**	**Estimate**	**p-value**	**SE**	**tStat**	**DF**
(Intercept)	-0.96152	0.52747	1.5165	-0.63405	102
PVE growth rate	-21.833	9.4472×10^{-18}	2.0947	-10.423	102
PVE lag time	1.4238	3.6464×10^{-33}	0.079734	17.857	102
PPU growth rate	-0.39277	0.91429	3.6405	-0.10789	102
PPU lag time	-0.23682	0.031472	0.10858	-2.1811	102
Starting cells ratio	0.58324	0.011955	0.22789	2.5593	102

The contribution of population kinetics and starting cell ratios to the stationary productivity ratio of *P. putida* (PPU) and *P. veronii* (PVE) in droplets (10 mM succinate) was assessed via a GLME analysis (as implemented by *fitglme* in MATLAB). SE gives the Standard Error for the estimated value (Estimate) of each Fixed effects coefficient. P-values from two-sided t-test. DF gives the Degree of Freedom corresponding to the t-statistic (tStat).

Supplementary Table 3. 21C growth medium composition

P. putida defined medium (Gerhard *et al.*, 1981):
(per liter)

C21 10X basis	100 ml
Hutner's vitamin free mineral base (sterile)	20 ml
vitamins (500 x) sterile	2 ml
Carbon substrate	x ml

Complete up to 1 L final volume with Millipore filtered water

C21 10X basis:
(per liter)

NH_4Cl	10 g
$Na_2HPO_4 \cdot 2H_2O$	34.9 g
KH_2PO_4	27.7 g

pH 6.8, sterilize by autoclaving

Hutner's vitamin free mineral base:
(per liter)

NTA (nitrilotriacetic acid, chelating agent)	10 g
$MgSO_4 \cdot 7H_2O$	14.45 g
$CaCl_2 \cdot 2H_2O$	3.33 g
$(NH_4)_6Mo_7O_{24} \cdot 4H_2O$	9.74 mg
$FeSO_4 \cdot 7H_2O$	99 mg
Metals 44	50 ml

sterilize by filtration and store at 4 °C.

Metals 44:
(per 100 ml)

$Na_4EDTA \cdot 4H_2O$	387 mg
$ZnSO_4 \cdot 7H_2O$	1.095 g
$FeSO_4 \cdot 7H_2O$	914 mg
$MnSO_4 \cdot H_2O$	154 mg
$CuSO_4 \cdot 5H_2O$	39.2 mg
$Co(NO_3)_2 \cdot 6H_2O$	24.8 mg
$Na_2B_4O_7 \cdot 10H_2O$	17.7 mg

+ a few trops of 6 N H_2SO_4
sterilize by filtration and store at 4 °C.

Vitamin solution (500 x):
(per 100 ml)

Biotin	0.5 mg
Nicotinic acid	50 mg
thiamin hydrochloride	25 mg

sterilize by filtration and store at 4 °C.

Supplementary Table 4. Strains and pre-culture procedures

Strain name	Agar plate media	Pre-culture media	Pre-culture time	Final OD_{600} (before mixing)	Fluorescent marker for identification	Source or reference	Gate in FCM
P. putida (PPU)	NA	21C MM + 10 mM succinate	16h	0.02	eGFP	Carraro et al. 2020 [1]	FSC-H, FITC-H
P. putida (PPU)	NA	21C MM + 10 mM succinate	16h	0.02	mCherry	This study	FSC-H, PE-TexasRed-H
P. veronii (PVE)	NA	21C MM + 10 mM succinate	16h	0.05	mCherry	Dubey et al. 2021 [2]	FSC-H, PE-TexasRed-H
Pseudomonas sp. Leaf15 (L15)	R2A	21C MM + 10 mM succinate	48h	0.02	mScarlet-I	Helfrich et al. 2018 [3], This study	FSC-H, PE-TexasRed-H
Sphingomonas wittichii (RW1)	R2A	21C MM + 4 mM salicylate	48h	0.02	eGFP	Coronado et al. 2015 [4]	FSC-H, FITC-H
P. protegens CHA0 (CHA0)	NA	21C MM + 10 mM succinate	16h	0.05	GFP2	Vacheron et al. 2021 [5]	FSC-H, FITC-H
P. protegens Pf-5 (Pf-5)	NA	21C MM + 10 mM succinate	16h	0.05	mScarlet-I	This study	FSC-H, PE-TexasRed-H

Supplementary Table 5. Strains and media conditions for each experiment

Experiment scenario	Strains involved	Cultivation media	Droplet sampling times	Droplet size tested	Starting cell density after mixing (OD_{600})	Droplet time-lapse
Substrate competition	PPU; PVE	MM C21 + 10 mM succinate	0, 24 h	40 µm	PPU: 0.01<br>PVE: 0.02	yes
Substrate competition	PPU; PVE	MM C21 + 10 mM succinate	0, 24 h	80 µm	PPU: 0.01<br>PVE: 0.02	no
Substrate competition	PPU; PVE	MM C21 + 10 mM succinate	0, 24 h	80 µm	PPU: 0.02<br>PVE: 0.04	no
Substrate independence	PPU; PVE	MM C21 + 10 mM d-mannitol + 6.67 mM putrescine	0, 24, 48 h	40 µm	PPU: 0.01<br>PVE: 0.02	no
Substrate competition	PPU (GFP); PPU (mCherry)	MM C21 + 10 mM succinate	0, 24 h	40 µm	0.01 for both	no
Substrate independence + growth inhibition	L15; RW1	MM C21 + 4 mM succinate + 1.5 mM salicylate	0, 17, 24, 48 h	40 µm	0.02 for both	no
Substrate competition + tailocin interaction	CHA0; Pf-5	MM C21 + 4 mM succinate	0, 24, 48 h	40 µm	0.025 for both	yes

Strain abbreviations: see Supplementary Table 4.

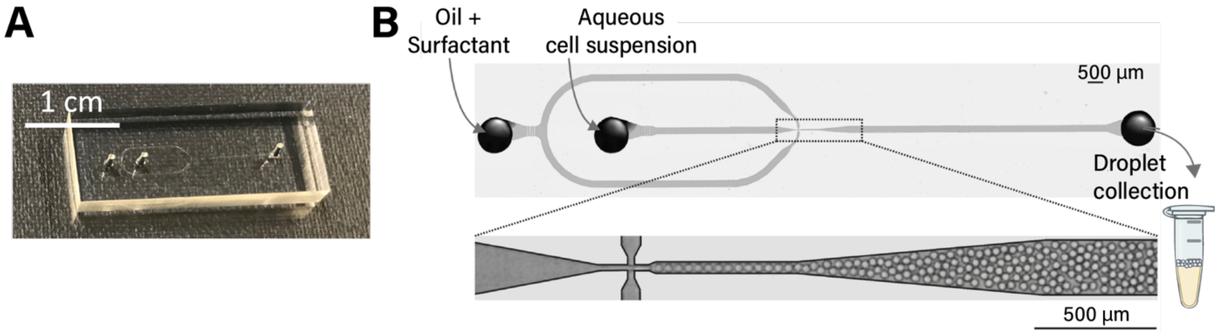

Supplementary Fig. 1: Encapsulation of cells in picoliter droplets by microfluidic operation. A) Microfluidic chip setup for producing microdroplets, according to Duarte *et al.* [6]. B) Schematic drawing of oil and cell suspension flow junctions. The junction between oil and aqueous flows has a 40 µm x 40 µm x 40 µm dimension, allowing for the generation of monodispersed 40 µm-sized droplets (as in the picture below). Droplets are directly collected in an Eppendorf tube. Picture courtesies: Achille Mariotti.

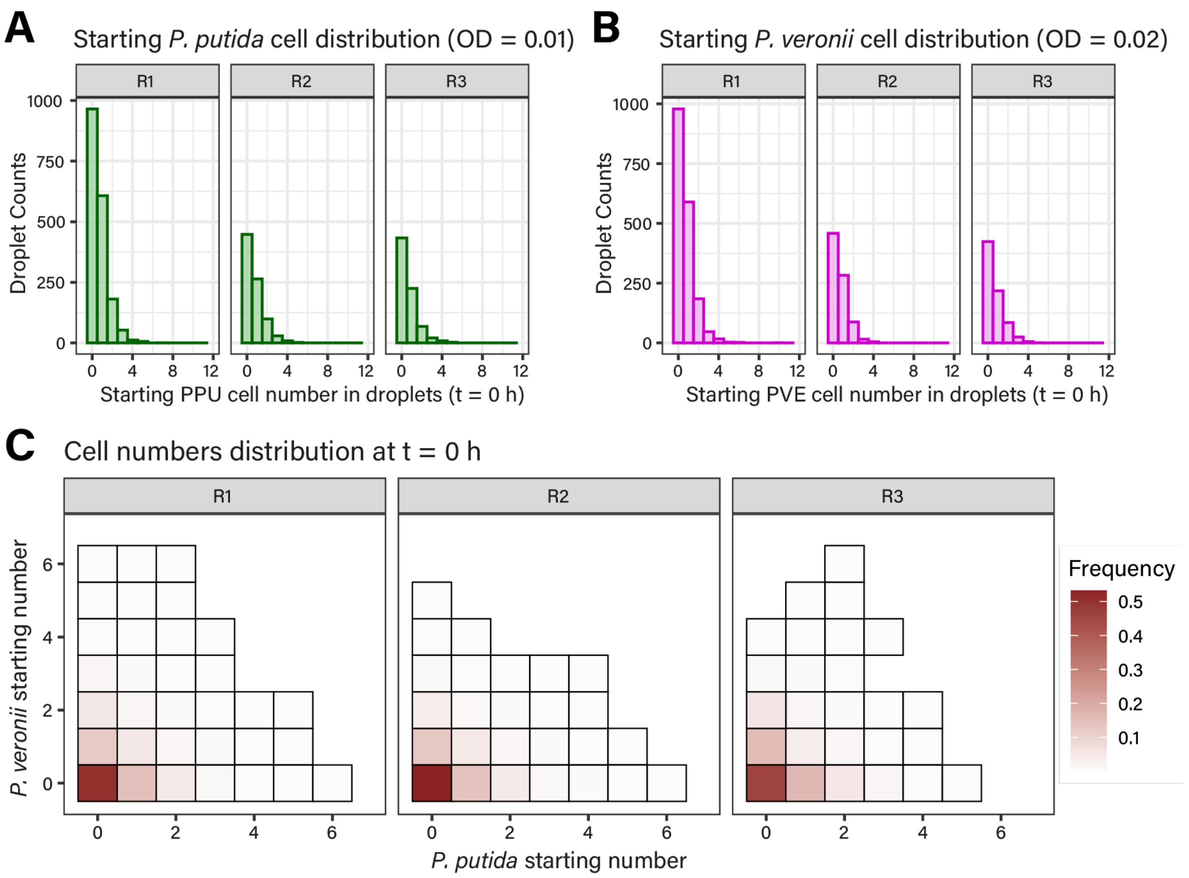

Supplementary Fig. 2: Starting cell distributions for *P. putida* and *P. veronii* after encapsulation in 35-pL volume droplets. A) Measured starting cell distributions for *P. putida* (PPU) and B) *P. veronii* (PVE) cells at the beginning of the succinate competition experiment in coculture droplets (t = 0 h, including *mix*, *solo* and *empty* droplets). C) Heatmap of paired frequency distributions in droplets (t = 0 h, n=3 biological replicates). Source data are provided as source data file.

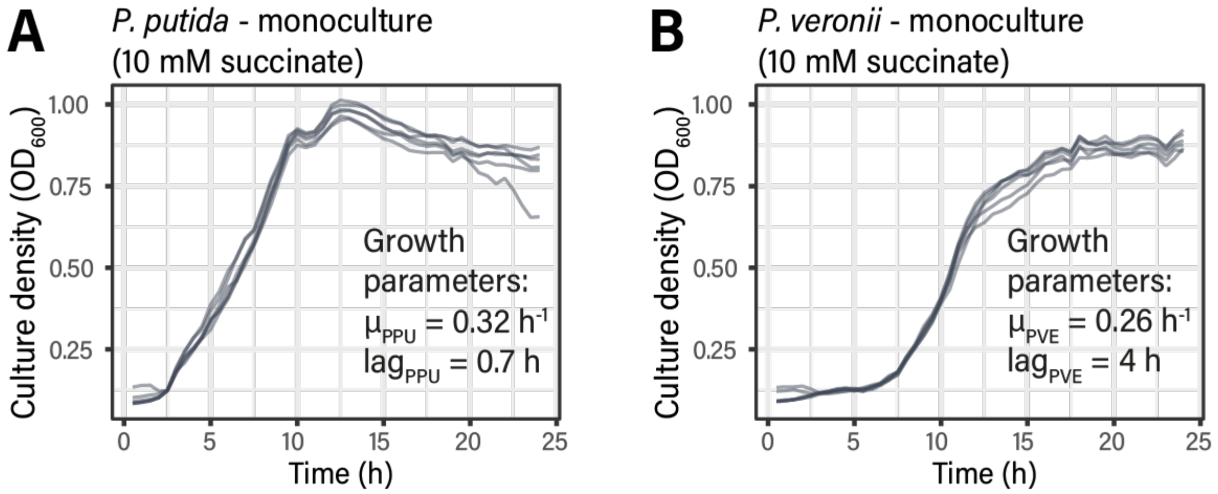

Supplementary Fig. 3. **Growth (as increase of culture turbidity) of *P. putida* or *P. veronii* in liquid suspended monocultures**. Growth curves of *P. putida* (A) and *P. veronii* (B) monocultures on 10 mM succinate in n = 7 replicates (individiual lines). Growth measured here as culture density increase (OD_{600}; same experiment as in Fig. 2a, which shows respective fluorescence values). Culture density increase was used to extract growth kinetic parameters (yield, growth rate and lag-time) of both species on succinate, by utilising the MCMC Metropolis Hasting algorithm[7]. Source data are provided as source data file.

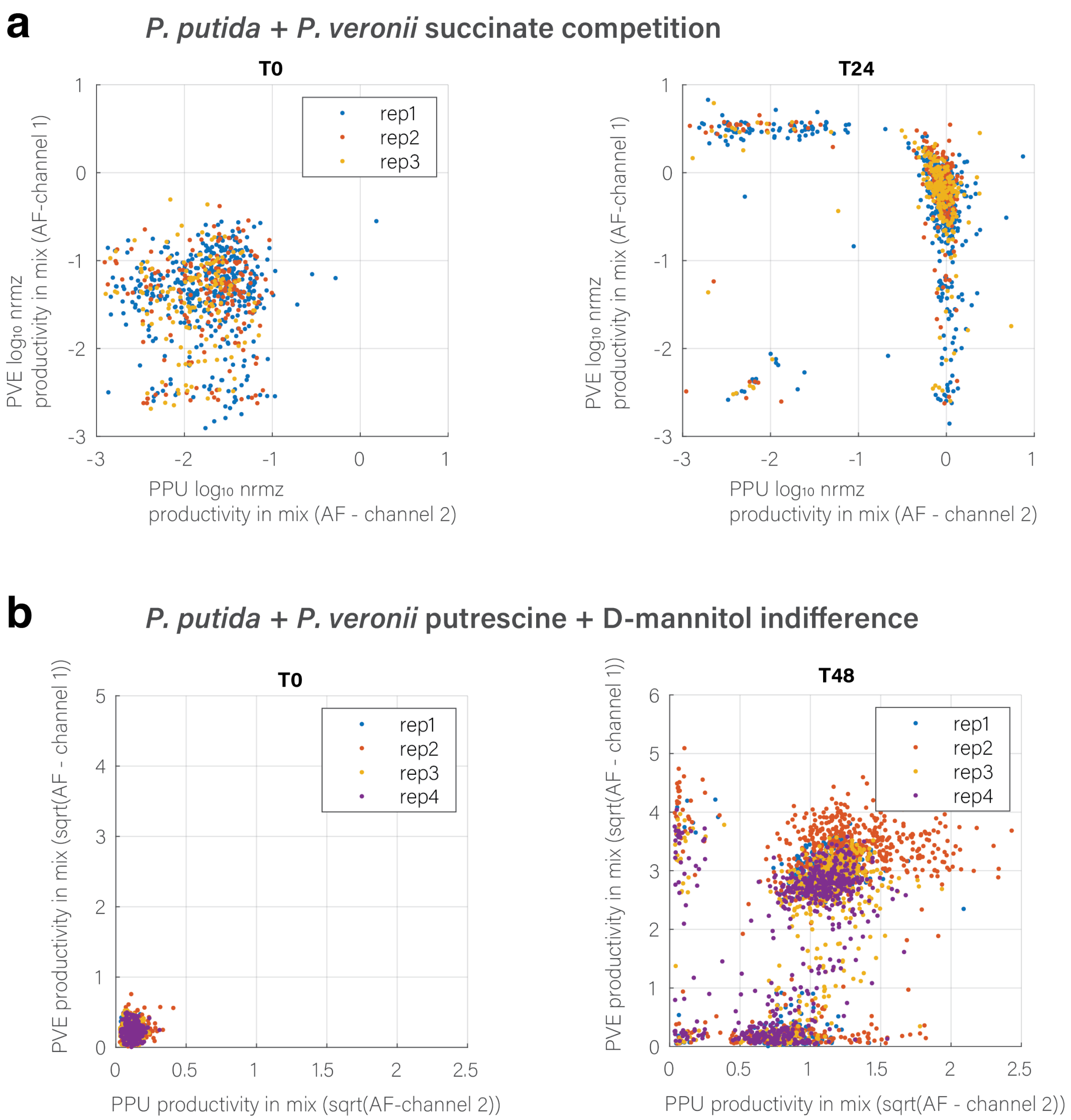

Supplementary Fig. 4. Comparison of T=0 and T24 or T48 h per droplet productivity (AF Area x fluorescence levels) for *P. putida* and *P. veronii*. Scatter diagrams show AF fluorescence x area-values for *mix* droplets (i.e., containing both strains) for each of the strains, at the start of the incubation and after 24 h (**a**) or 48 h (**b**) incubation in emulsions. Each dot is a measurement of a single droplet. Colors are from independent emulsion replicates. AF-values expressed as $\log_{10}$ of the median-normalized fluorescence channel specific for each strain (i.e., channel 2 = *P. putida* or PPU; channel 1 = *P. veronii* or PVE), respective to t=24 h sampling point (**a**), or as square-root transformed AF (**b**). (**a**) Substrate competition with succinate. (**b**) Substrate indifference with D-mannital and putrescine. Note how the t=0 values are suggestive for dormant or growth-compromised cells at the later stage. Source data are provided as source data file.

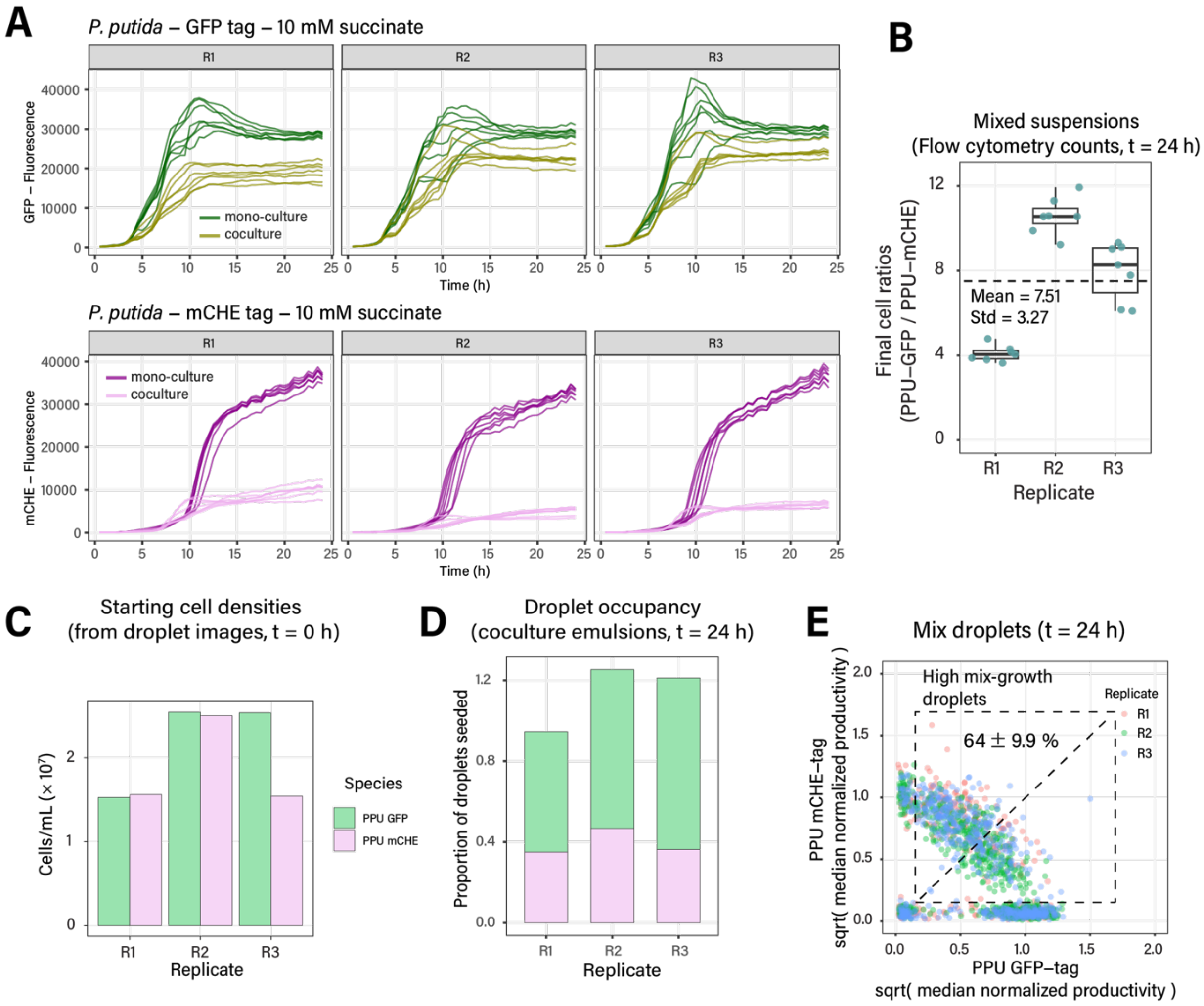

Supplementary Fig. 5. Droplet growth outcomes in an isogenic *P. putida* co-culture control on 10 mM succinate. A) Growth (as strain-specific fluorescence) on 10 mM succinate in 96-well plates of isogenic *P. putida* tagged either with constitutively expressed GFP or mCherry, in monocultures (darker colors) or coculture (lighter colors, n = 7 technical replicates per biological replicate; R1–R3). **B**) Strain-specific cell counts after 24 h by flow cytometry, shown as ratio of GFP- vs mCherry-labeled strain (dots are technical replicates). Note the difference in fitness effect of the labels. **C**) Mean starting cell densities of each of the strains in droplets, estimated from droplet images (here converted to cells per ml with a 35-pL droplet volume). Note that strains were encapsulated in droplets from the same suspension as used for the liquid 96-well plate cultures. **D**) Proportion of imaged droplets after 24 h with detected GFP- or mCherry-signal (solo and mix droplets combined). Slightly more droplets were inoculated with GFP-labeled *P. putida* cells than their mCherry-isogenic variant. **E**) Paired-productivity (as square-root transformed median-normalized AF signal) across mix droplets (i.e., carrying both fluorescent signals) after 24 h. Dashed-line box highlights droplets where both strains grew together, excluding potentially growth-impaired cells. The diagonal dashed line indicates the expected equivalent productivity of either of the strains. Note how substrate competition here is a more homogeneous outcome for either of the strains. Source data are provided as source data file.

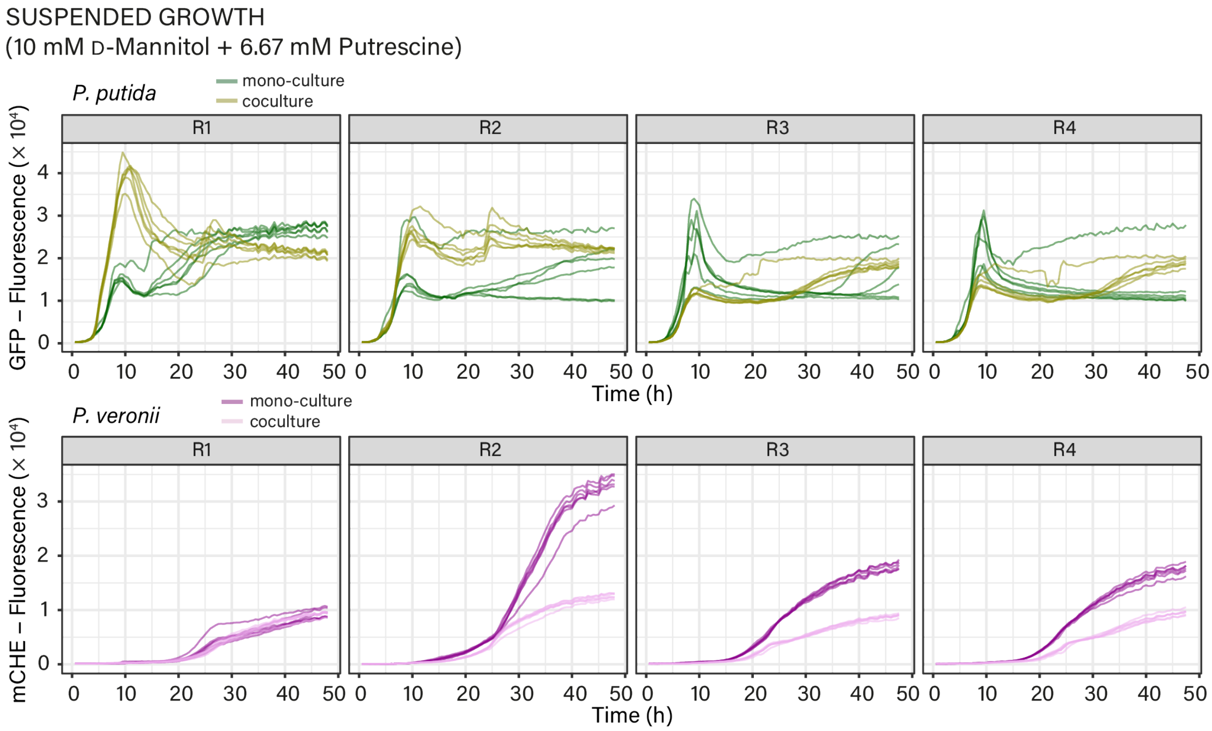

Supplementary Fig. 6. Growth curves of *P. putida* and *P. veronii* in liquid culture on D-mannitol and putrescine. Plots show the strain-specific fluorescence signal increase measured in n=6 technical replicates in 96-well plates of mono- or cocultures on both substrates simultaneously (n = 4 biological replicates; R1-R4). Source data are provided as source data file.

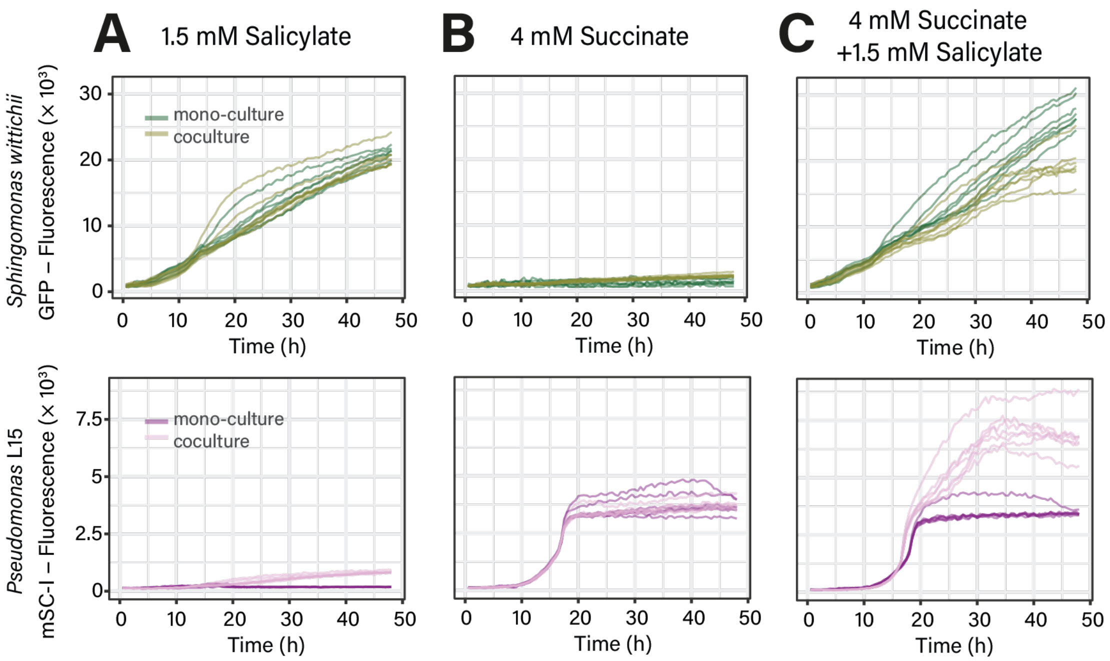

Supplementary Fig. 7. Growth curves of *Pseudomonas* L15 and *Sphingomonas wittichii* RW1 in liquid culture on succinate or salicylate. Displays show the strain-specific fluorescence values for each of the strains grown in mono- or coculture in 96-well plates (n=7 technical replicates). **A**) Salicylate, **B**) succinate, or **C**) both salicylate and succinate. Note the independent substrate utilisation of salicylate by RW1 and of succinate by L15, and a potential cross-feeding from RW1 to L15 on salicylate. Source data are provided as source data file.

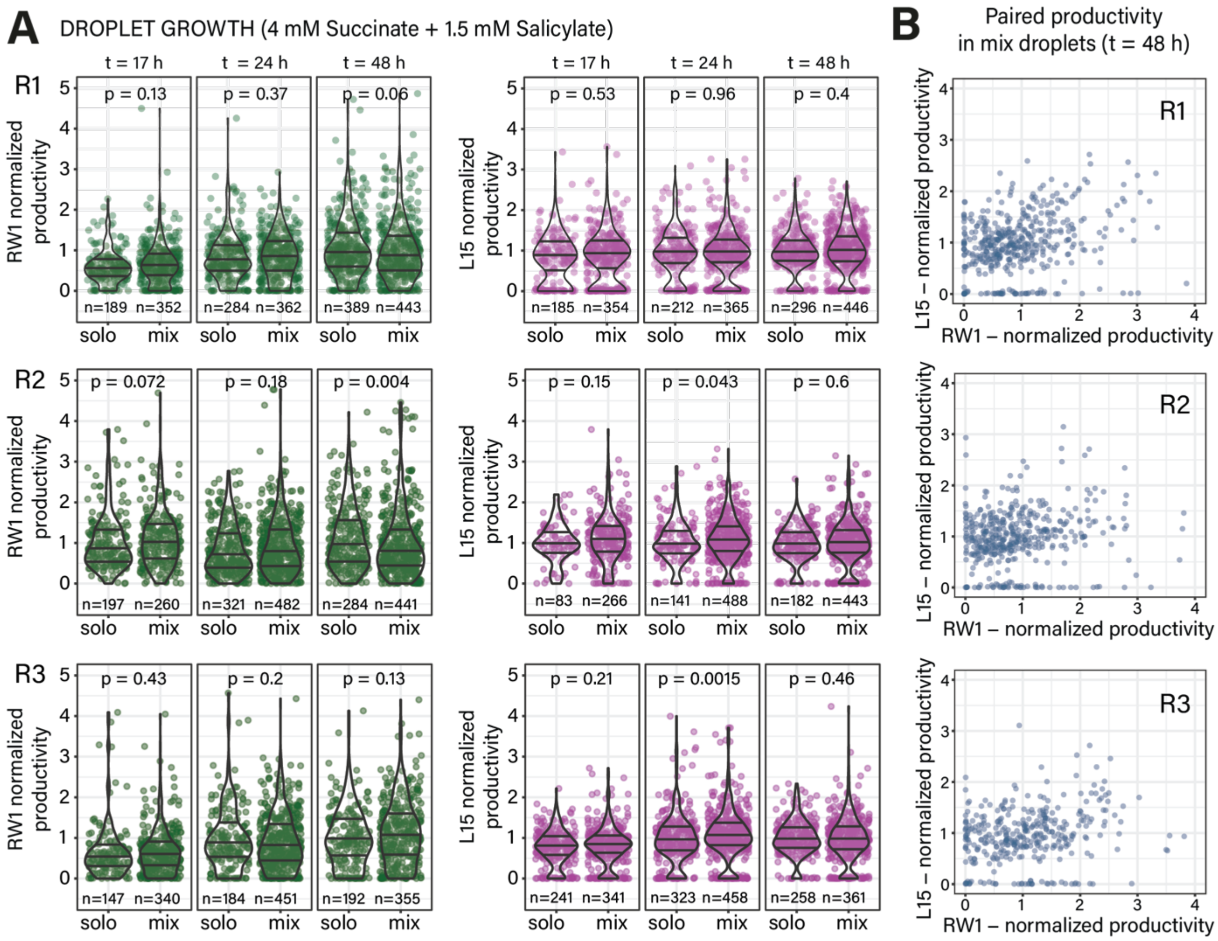

Supplementary Fig. 8. Individual replicates of productivities of *Pseudomonas* Leaf15 and *Sphingomonas wittichii* RW1 in pL-droplets sampled at different incubation times. A) Productivities (as AF-values normalized to the replicate-specific median of the solo droplets at t=48) of either strain in solo and mix droplets (i.e., with both fluorescent signals detected) in incubations with salicylate and succinate. P-values from two-sided Wilcoxon rank sum tests. Droplet counts indicated within each jitter-plot. **B**) Paired productivities of L15 and RW1 in mix droplets after 48 h (same AF-value normalization as in panel **A**). R1, R2 and R3 denote the three biological replicates of the experiment. Source data are provided as source data file.

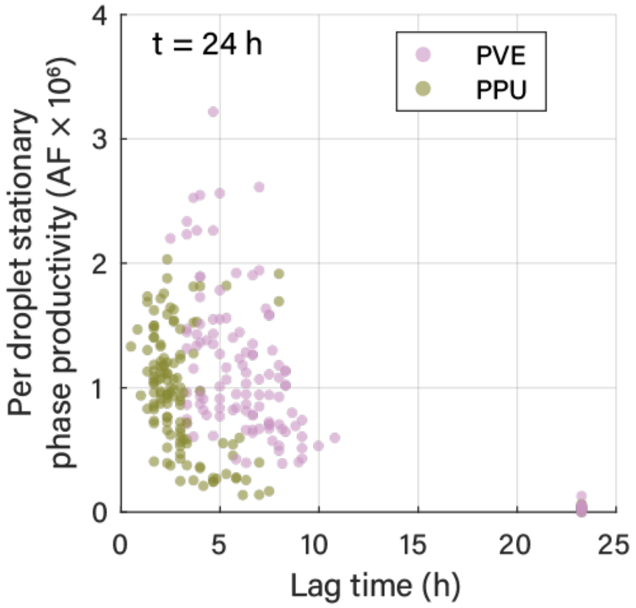

Supplementary Fig. 9. Stationary phase productivity of *P. putida* and *P. veronii* in mix droplets as a function of lag time. Data show strain-specific per-droplet AF-values after 24 h from time-lapse imaging of mix droplets growing on succinate (i.e., containing both *P. putida*; PPU and *P. veronii*; PVE), as a function of the observed lag time. Each dot is a measurement from a single droplet. Note the tendency that longer lag times result in low stationary phase productivity for either of the stains under competition. Source data are provided as source data file.

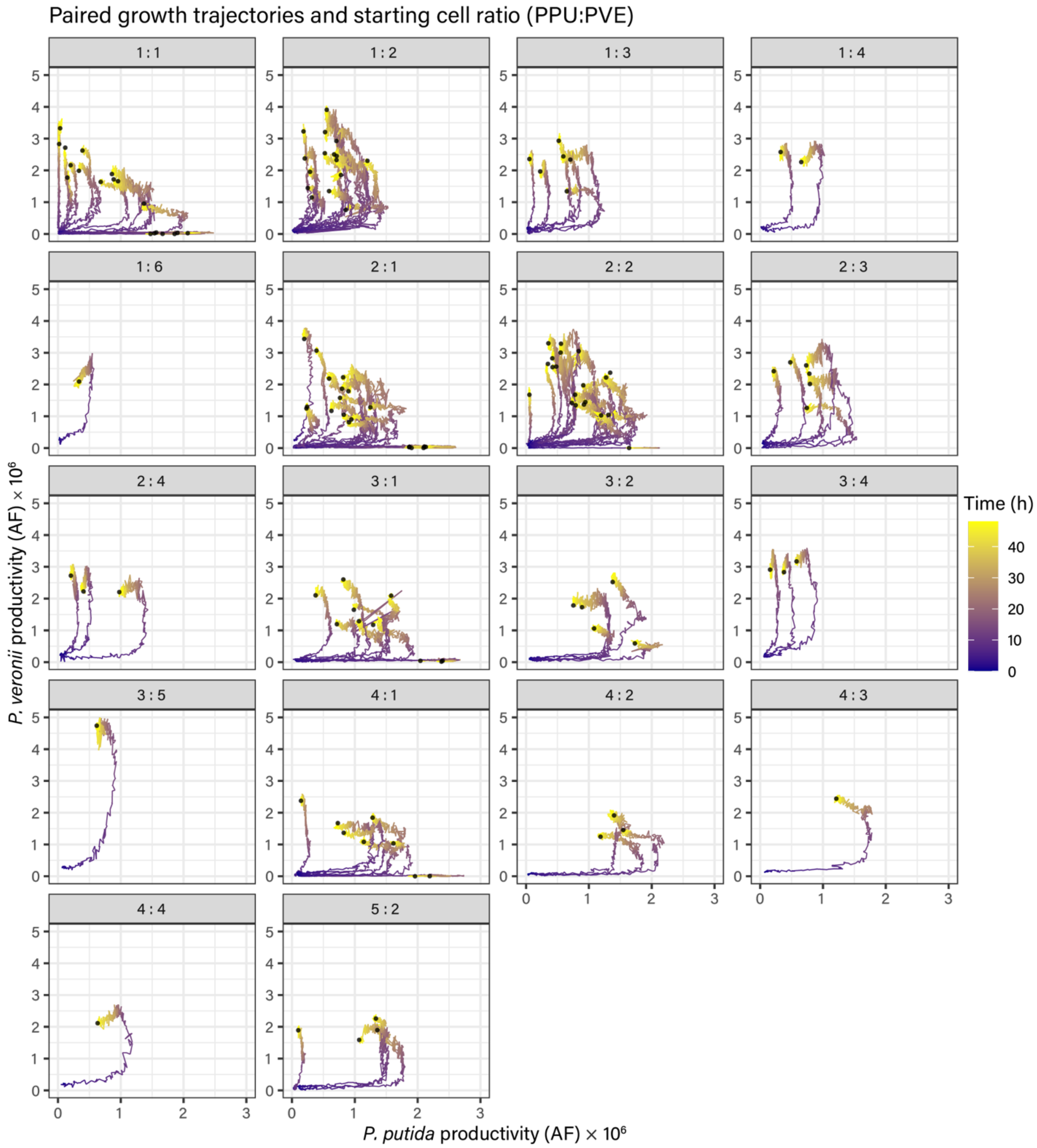

Supplementary Fig. 10. Paired growth trajectories of *P. putida* and *P. veronii* in time-lapse imaged droplets. Facets indicate individual droplet trajectories of strain-specific AF-signals over time (as color per the legend). The starting number of *P. putida* (PPU) and *P. veronii* (PVE) cells are denoted on the top of each facet (as the ratio of PPU:PVE cell numbers), regrouping paired growth curves of droplets starting at the same cell numbers. Source data are provided as source data file.

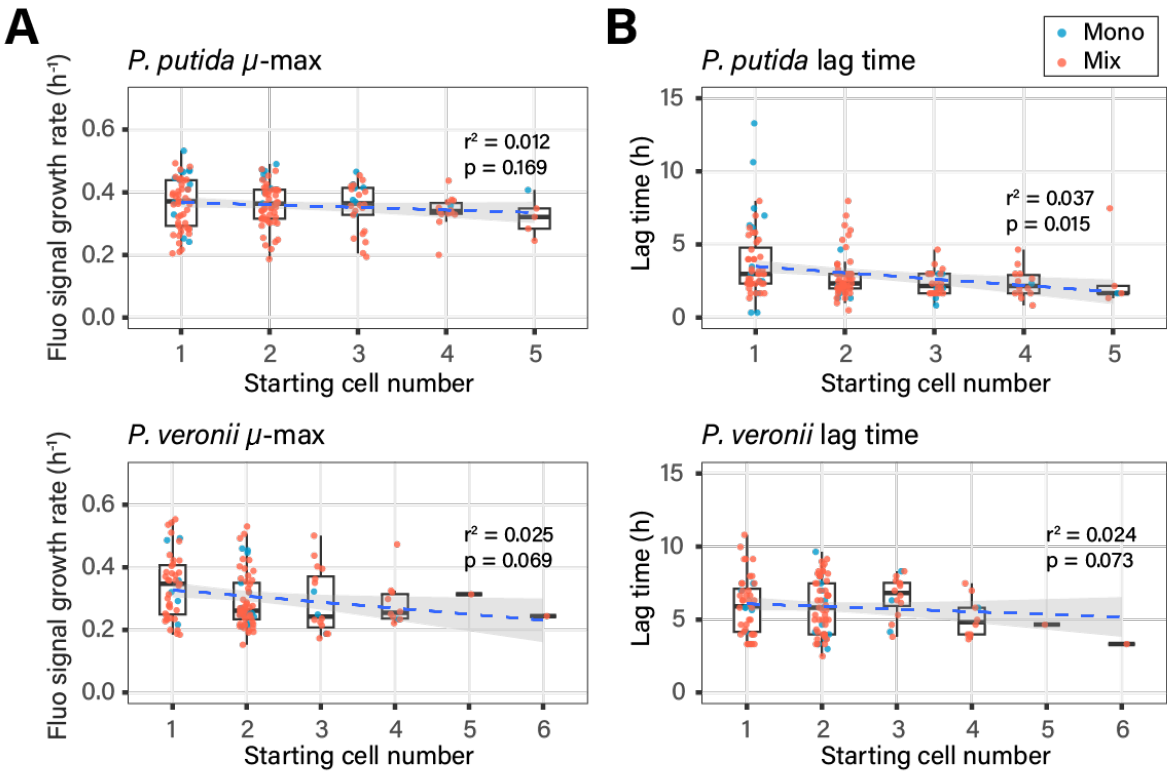

Supplementary Fig. 11. Effects of droplet starting cell numbers on observed population growth kinetics. A) Growth rates and **B**) lag times of *P. putida* and *P. veronii* in mono or mix droplets during time-lapse imaging, inferred from the strain-specific AF-fluorescence signal development, plotted as a function of the observed starting cell numbers for each species and per droplet. Each dot is a measurement from a single droplet (blue, mono; salmon, mix droplets). P-values from the comparison of slopes of the linear regression lines (r^2) between mono and mix droplets. Source data are provided as source data file.

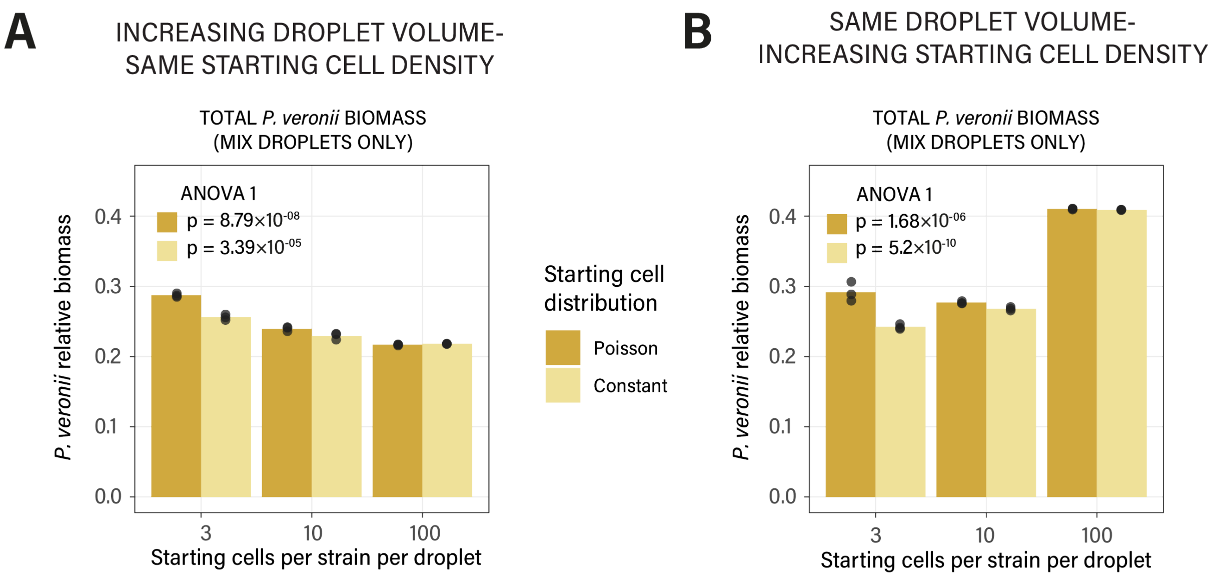

Supplementary Fig. 12. Simulated effects of increasing starting cell numbers on *P. veronii* relative abundance at stationary phase (mix droplets only). A) Total *P. veronii* relative biomass abundance compared to *P. putida* from mix droplets only, in simulated droplet growth (n= 500 droplets, 3 replicate simulations) with increasing founder population sizes of both *P. veronii* and *P. putida* in proportionally increased droplet volumes (hence, same starting cell density). Two starting conditions: Poisson-sampled or constant cell numbers. **B)** Simulations as in **A** but with constant droplet volume and increasing founder population sizes (hence: increasing starting cell densities). Since solo droplets are not accounted for, this simulation predicts a positive effect of increasing the degree of fragmentation on *P. veronii*'s overall abundance under competitive growth with *P. putida*. Bars indicate the mean of n = 3 replicate simulations, with individual data points indicated. P-values from ANOVA. Source data are provided as source data file.

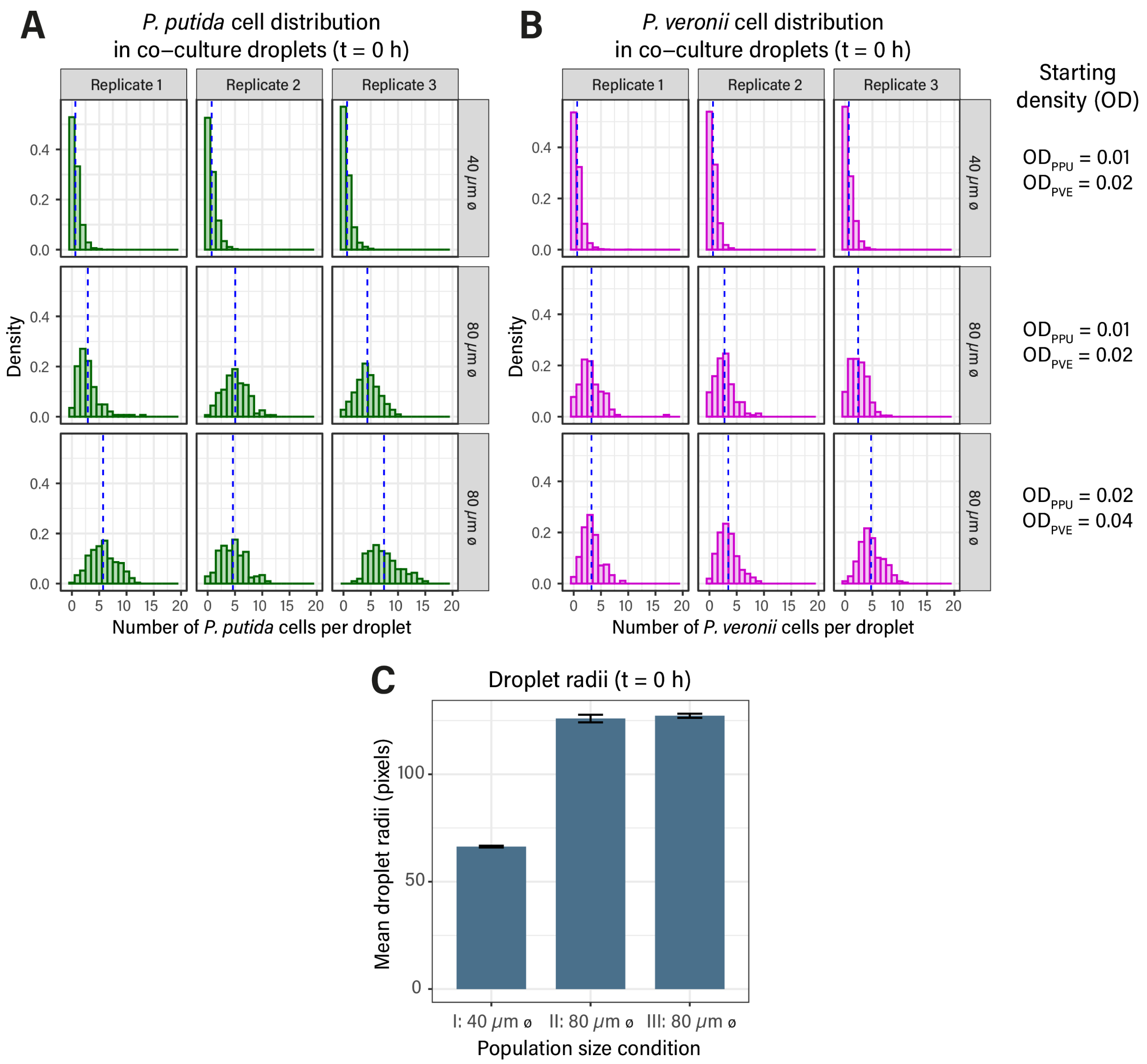

Supplementary Fig. 13: Measured per droplet starting cell distributions at higher intended founder cell population sizes of *P. putida* and *P. veronii*. Distributions of droplet cell numbers of **A**) *P. putida* and **B**) *P. veronii* at incubation start, inferred from droplet imaging (all droplets are taken into account, both empty, solo and mix). Dashed lines indicate the mean of observed cell numbers. **C**) Mean droplet radii in pixels for the different population size conditions (as illustrated in Fig. 8g). Error bars indicate standard deviation from the mean (n = 3 replicates). Source data are provided as source data file.

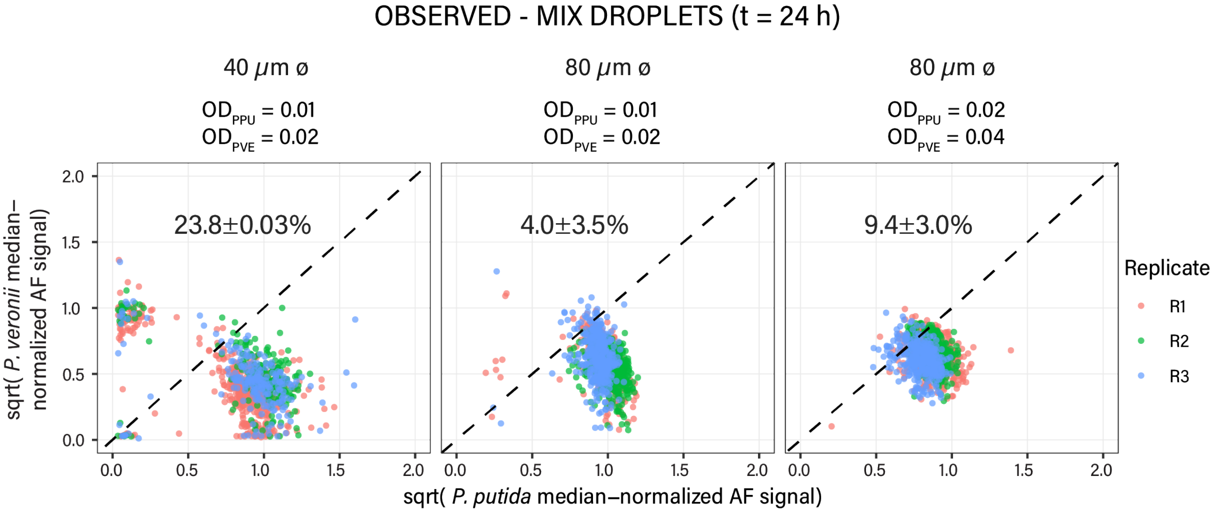

Supplementary Fig. 14: Strain-specific productivity scatter plots for mix droplets at increasing starting cell population sizes of *P. putida* and *P. veronii*. Plots show the square-root transformed median-normalized strain-specific productivity (as AF, or area x fluorescence) at t=24 h incubation under succinate competition of *P. putida* and *P. veronii* in mix droplets (n = 3 replicates; R1–R3). From left to right, increasing starting cell population sizes, as explained in Fig. 8. Percentages indicate the proportion of droplets within the diagonal zone above the dashed line (dominated by *P. veronii* when taking the normalized AF-productivity value into account). Note how the dispersal of per droplet values decreases at higher starting cell population sizes. Source data are provided as source data file.

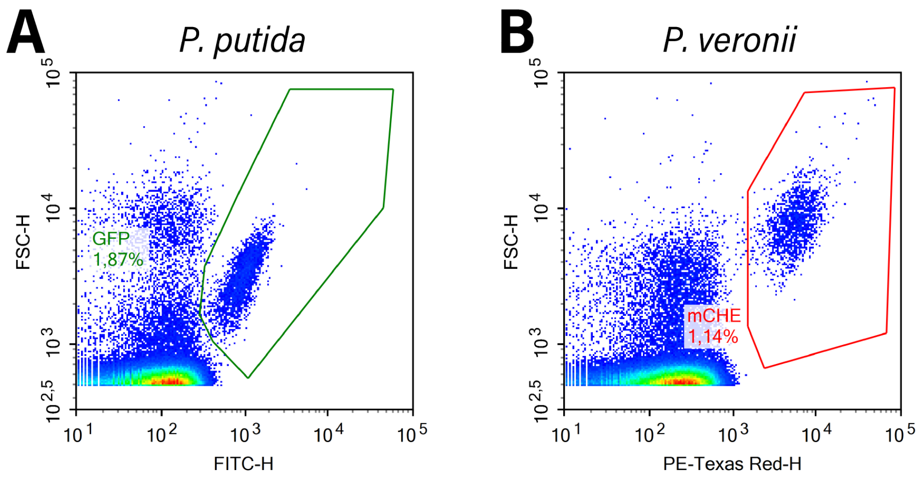

Supplementary Fig. 15: Gating strategy for strain-specific fluorescence detection in flow-cytometry. Examples for *P. putida* (A) and *P. veronii* (B). FSC-H, forward scatter signal. FITC-H, emission and detector settings for the GFP signal. PE-Texas Red-H, emission and detector settings for the mCherry signal.

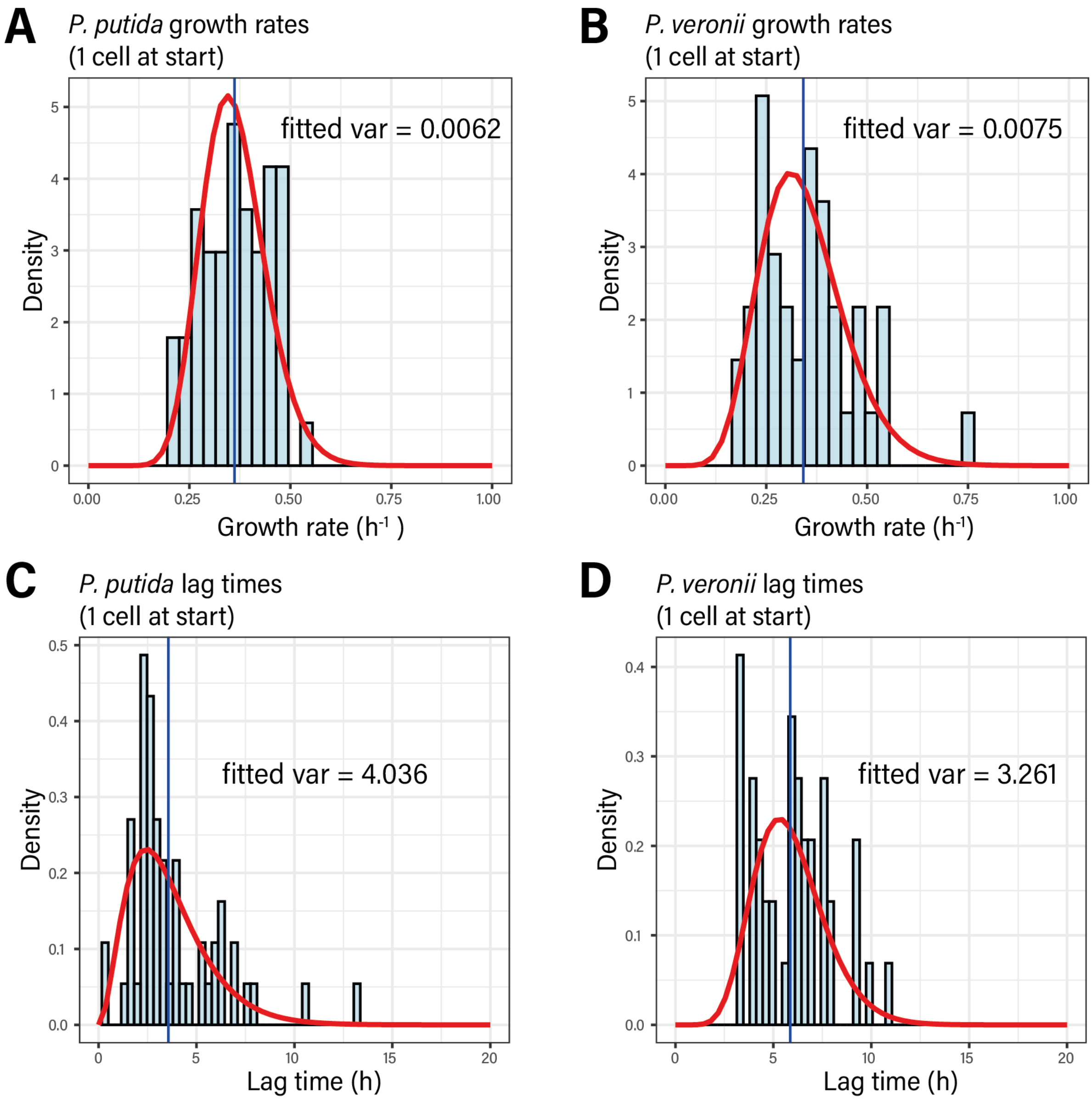

Supplementary Fig. 16. Distributions of growth kinetic parameters inferred from droplets with a single founder cell of *P. putida* or *P. veronii*. Growth rate (**A**) and lag time distributions (**C**) of *P. putida* and *P. veronii* (**B** and **D**), deduced from time-lapse imaged droplets with a single starting cell of either species (combining mono and solo droplets). Red line is the fitted Gamma distribution, with the calculated fitted parameter variance. Blue line is the fitted mean. Source data are provided as source data file.

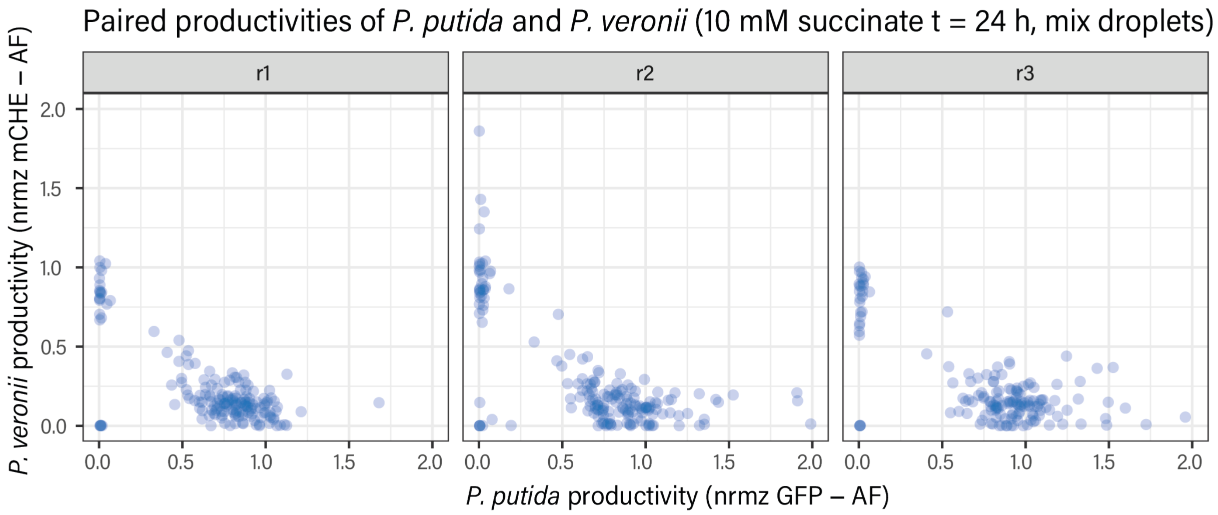

Supplementary Fig. 17. Reproducibility of droplet cultivation procedures. Diagrams show paired productivities of *P. putida* and *P. veronii* after 24 h in a triplicate emulsion (r1–r3) generated from a single suspension with 10 mM succinate. Productivities plotted as median-normalized strain-specific AF (Area x fluorescence); each dot is a measurement from an individual droplet. Source data are provided as source data file.

Supplementary References

1. Carraro, N., Richard, X., Sulser, S., Delavat, F., Mazza, C. & van der Meer, J. R. An analog to digital converter controls bistable transfer competence development of a widespread bacterial integrative and conjugative element. *Elife* **9** (2020).
2. Dubey, M., Hadadi, N., Pelet, S., Carraro, N., Johnson, D. R. & van der Meer, J. R. Environmental connectivity controls diversity in soil microbial communities. *Communications Biology* **4** (2021).
3. Helfrich, E. J. N. *et al.* Bipartite interactions, antibiotic production and biosynthetic potential of the Arabidopsis leaf microbiome. *Nat Microbiol* **3**, 909-919 (2018).
4. Coronado, E., Valtat, A. & van der Meer, J. R. *Sphingomonas wittichii* RW1 gene reporters interrogating the dibenzofuran metabolic network highlight conditions for early successful development in contaminated microcosms. *Environ Microbiol Rep* **7**, 480-488 (2015).
5. Vacheron, J., Heiman, C. M. & Keel, C. Live cell dynamics of production, explosive release and killing activity of phage tail-like weapons for *Pseudomonas* kin exclusion. *Commun Biol* **4**, 87 (2021).
6. Duarte, J. M., Barbier, I. & Schaerli, Y. Bacterial Microcolonies in Gel Beads for High-Throughput Screening of Libraries in Synthetic Biology. *ACS Synth Biol* **6**, 1988-1995 (2017).
7. Guex, I., Mazza, C., Dubey, M., Batsch, M., Li, R. & van der Meer, J. R. Regulated bacterial interaction networks: A mathematical framework to describe competitive growth under inclusion of metabolite cross-feeding. *PLoS Comput Biol* **19**, e1011402 (2023).
