# Supplementary material for: Fragmented micro-growth habitats present opportunities for alternative competitive outcomes
Source: Nat Commun. 2024 Aug 31;15:7591. doi: 10.1038/s41467-024-51944-z (PMC11365936; doi:10.1038/s41467-024-51944-z)
Supplement: Supplementary file 3 — Supplementary movie legends [file 41467_2024_51944_MOESM3_ESM.docx]

Supplementary Movie 1: Time-lapse compiled images of a single droplet with growing *P. protegens* CHA0 (green) and Pf-5 (magenta) on 10 mM succinate.

Supplementary Movie 2: Time-lapse compiled images of a single droplet with growing *P. protegens* CHA0 (green) and Pf-5 (magenta) on 10 mM succinate, followed by Pf-5 lysis – assumed as a result of CHA0-released tailocins.
